# Retraction: Phase 2 Assessment of a New Functional Pain Scale by Comparing It to Traditional Pain Scales

**DOI:** 10.7759/cureus.r58

**Published:** 2022-06-08

**Authors:** Harris W Thomas, Adeolu A Adeboye, Rachel Hart, Harshavardhan Senapathi, Michael Hsu, Sneha Singh, Tejaswini Maganti, Victor Kolade, Abistanand Ankam, Amlish Gondal

**Affiliations:** 1 Pain Management, Guthrie Robert Packer Hospital, Sayre, USA; 2 General Surgery, Guthrie Robert Packer Hospital, Sayre, USA; 3 Trauma and Acute Care Surgery, Guthrie Robert Packer Hospital, Sayre, USA; 4 Anesthesia, Guthrie Robert Packer Hospital, Sayre, USA; 5 Internal Medicine, Guthrie Robert Packer Hospital, Sayre, USA

This article has been retracted at the request of the authors due to a mistakenly identifying the scale used as the Wong-Baker FACES Pain Scale, when in fact it was the Universal Pain Assessment Tool that was used. Given how heavily this scale is featured within the article, the decision was made to retract after consulting with the Wong-Baker FACES Foundation and Cureus editorial staff. The authors regret the error and any confusion it may have caused.

